# Interprofessional Education Through Case Study: Learners’ Attitudes on Interprofessional Education

**DOI:** 10.1093/geroni/igaa057.3141

**Published:** 2020-12-16

**Authors:** Anne Halli-Tierney, Rebecca Allen, Dana Carroll, Robert McKinney

**Affiliations:** 1 The University of Alabama, Tuscaloosa, Alabama, United States; 2 Alabama Research Institute on Aging, Tuscaloosa, Alabama, United States; 3 Auburn University Harrison School of Pharmacy, Tuscaloosa, Alabama, United States

## Abstract

Interprofessional education case sessions allow learners to apply discipline-specific knowledge to real-life scenarios through thorough facilitated discussion of a patient case. Our interprofessional case discussion was implemented for learners to develop care plans for complex geriatric patients; learners have intentional time to learn with, from and about each other’s roles in geriatric care. All learners receive the case and work through it from their discipline’s perspective, then join a facilitated group discussion to develop collaborative care plans. Participants were surveyed using the ICAS and qualitative comments about perceptions of interprofessional learning, and most interprofessional (medicine, pharmacy, psychology and social work) learners found the sessions to be educational. Themes emerging from qualitative analysis about what was most educational were “different professional approaches”, “professional roles”, “collaboration” and “problem solving”. Typically, learners were unable to identify “least educational” components to the activity. Overall feedback from learners aligns with the goals of interprofessional education. Part of a symposium sponsored by the Mental Health Practice and Aging Interest Group.

